# Patients with encrusted ureteral stents can be treated by a single session combined endourological approach

**DOI:** 10.1590/S1677-5538.IBJU.2020.0511

**Published:** 2020-12-20

**Authors:** Roberto Iglesias Lopes, Rodrigo Perrella, Carlos Hirokatsu Watanabe, Fabricio Beltrame, Alexandre Danilovic, Claudio Bovolenta Murta, Joaquim Francisco de Almeida Claro, Fabio Carvalho Vicentini

**Affiliations:** 1 Hospital Brigadeiro Divisão de Urologia São PauloSP Brasil Divisão de Urologia, Hospital Brigadeiro, São Paulo, SP, Brasil; 2 Universidade de São Paulo Faculdade de Medicina Hospital das Clínicas São PauloSP Brasil Divisão de Urologia, Hospital das Clínicas, Faculdade de Medicina da Universidade de São Paulo, SP, Brasil

**Keywords:** Stents, Ureter, Nephrolithotomy, Percutaneous

## Abstract

**Purpose:**

To describe our experience in the management of retained encrusted ureteral stents using a single session combined endourological approach.

**Materials and Methods:**

Patients with retained encrusted ureteral stents who had been submitted to a single session combined endourological approach from June 2010 to June 2018 were prospectively evaluated. Patients were divided according to the Forgotten-Encrusted-Calcified (FECal) classification. The stone burden, surgical intervention, number of interventions until stone free status, operation time, hospital stay, complications, stone analysis, and stone-free rate were compared between groups. ANOVA was used to compare numerical variables, and the Mann-Whitney or Chi-square test to compare categorical variables between groups.

**Results:**

We evaluated 50 patients with a mean follow-up of 2.9±1.4 years (mean±SD). The groups were comparable in terms of age, sex, laterality, BMI, comorbidities, ASA, reason for stent passage, and indwelling time. The stone burden was higher for grades IV and V (p=0.027). Percutaneous nephrolithotomy was the most common procedure (p=0.004) for grades IV and V. The number of procedures until the patients were stone-free was 1.92±1.40, and the hospital stay (4.2±2.5 days), complications (22%), and stone analysis (66% calcium oxalate) were similar between groups. The stone-free rate was lower in grades III to V (60%, 54.5%, and 50%).

**Conclusions:**

The endoscopic combined approach in the supine position is a safe and feasible technique that allows removal of retained and encrusted stents in a single procedure. The FECal classification seems to be useful for surgical planning.

## INTRODUCTION

Ureteral stents have been widely utilized since 1967, following open or endoscopic ureteral surgery, ureteral strictures, ureteropelvic junction obstructions, malignancies, or treatment of urinary stones (1). Encrusted and retained ureteral stents represent the most challenging complication associated with ureteral stents (2, 3). Management is difficult, and several procedures might be necessary (2, 4-7).

A modification of the original Valdivia supine position (Galdakao-modified Valdivia positioning) has been proposed to perform percutaneous nephrolithotomy (PCNL) (8), and allows the combined use of simultaneous retrograde and percutaneous access to the urinary tract, while preserving the surgical and anesthesiologic advantages of supine position (9-11). The term endoscopic combined intra-renal surgery (ECIRS) has been applied to this approach (12). Although several endourologic methods have been reported for the management of encrusted and retained stents, there is no formal recommendation in urology guidelines.

The Forgotten-Encrusted-Calcified (FECal) classification was created to predict outcomes when treating these patients, and it seems to provide an alternative for the surgical management dilemma. However, there are currently no data to advise patients on expected surgical outcomes or surgical planning (3).

In this article, we describe our experience in the management of retained encrusted stents using an endoscopic combined intra-renal surgery and provide useful insight into this difficult condition.

## MATERIALS AND METHODS

**Figure 1:**
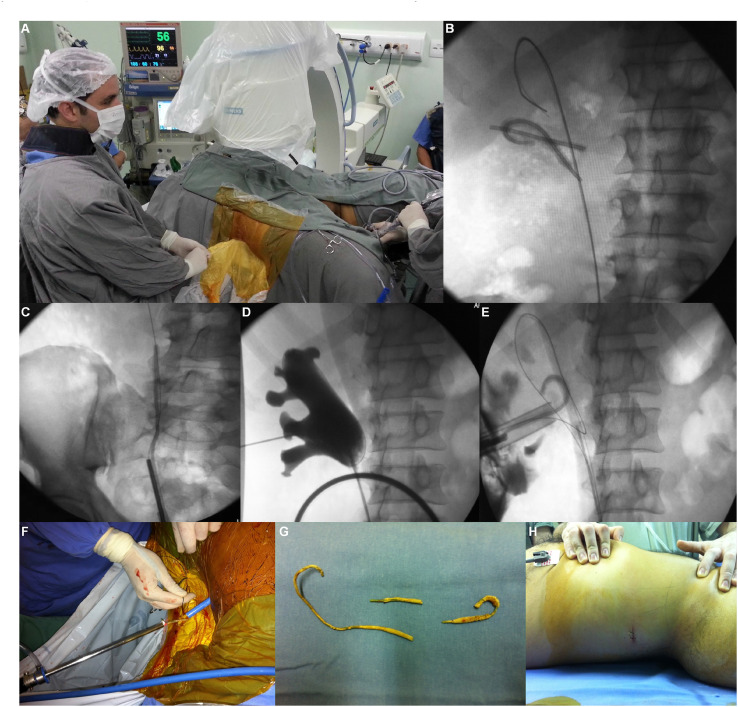
Image series showing combined approach with Galdakao-modified Valdivia position for removal of encrusted fragmented stent. A) Galdakao-modified Valdivia position. Percutaneous access under biplanar fluoroscopic guidance. B) Guidewire placed retrogradely in the upper calyx. Note two stent fragments located in the kidney. C) Semirigid ureteroscopy. Notice distal stent fragment. D) Retrograde pyelography with 6 Fr ureteral catheter placed into the pelvis and lower calyx puncture. E) Stent fragments retrieval with stone grasping forceps. F) Encrusted stent fragment removal through Amplatz® sheath. G) Completely encrusted stent fragmented in three parts. H) Incision after percutaneous access.

From June 2010 to June 2018, all patients with retained and encrusted ureteral stents were submitted to a combined endourological approach with Galdakao-modified Valdivia positioning (Figure-1). Ethics committee institutional approval and informed consent for the enrolled patients were obtained before treatment. Encrusted stent was defined as one that could not be removed at the first attempt using gentle manual traction with grasping forceps.

The ureteral stent encrustation was defined as was evaluated at the first clinical visit by abdominal plain radiography, and patients were referred within 1 week for definitive treatment. Preoperative assessment included patient age and sex, body mass index (BMI), average stent indwelling, reason for stent placement, and American Society of Anesthesiologists Physical Status Classification System (ASA). Abdominal and pelvic computed tomography (CT), both plain and with intravenous ionized contrast, was performed in all cases to evaluate renal function and to assess the location and size of the stent calcifications (stone burden) for surgical management. Stone burden was estimated using an ellipsoid formula (stone volume=π.l.w.d.0.167), where length (l), width (w), and depth (d) are the stone diameters measured in three axes. Renal function assessment was completed with renal scintigraphy. In non-functioning kidneys (less than 10% function on scintigraphy) laparoscopic total nephrectomy was performed, and patients were excluded from the study. Groups were divided by the assistant urologist according to a previously reported calcified stent scale (FECal classification) (3), as follows:

Grade I - Minimal linear encrustations along either portion;

Grade II - Circular encrustation completely encasing either portion;

Grade III - Circular encrustation completely encasing either portion or linear encrustation of the ureteral aspects;

Grade IV - Circular encrustations completely encasing both portions;

Grade V - Diffuse and bulky encrustations completely encasing the proximal, distal, and ureteral portions.

Urine samples were collected before surgery and infections were treated using antibiotic susceptibility profiles. In cases of a negative culture, a preoperative prophylactic antibiotic course, with 100mg nitrofurantoin, was administered orally once a day starting 7 days before surgery (13). All procedures were performed by the same surgeon (R.I.L.), who was experienced in prone and supine percutaneous nephrolithotomy (PCNL), retrograde ureterolithotripsy (URS), and retrograde intrarenal surgery (RIRS). Surgical management was based on renal function, location, and the stone burden (considering encrusted stents and associated stones).

## SURGICAL TECHNIQUE

Galdakao-modified Valdivia positioning was used in all cases with a functioning kidney, and a combined endourological approach was performed simultaneously as shown in Figure-1.

**Figure 2:**
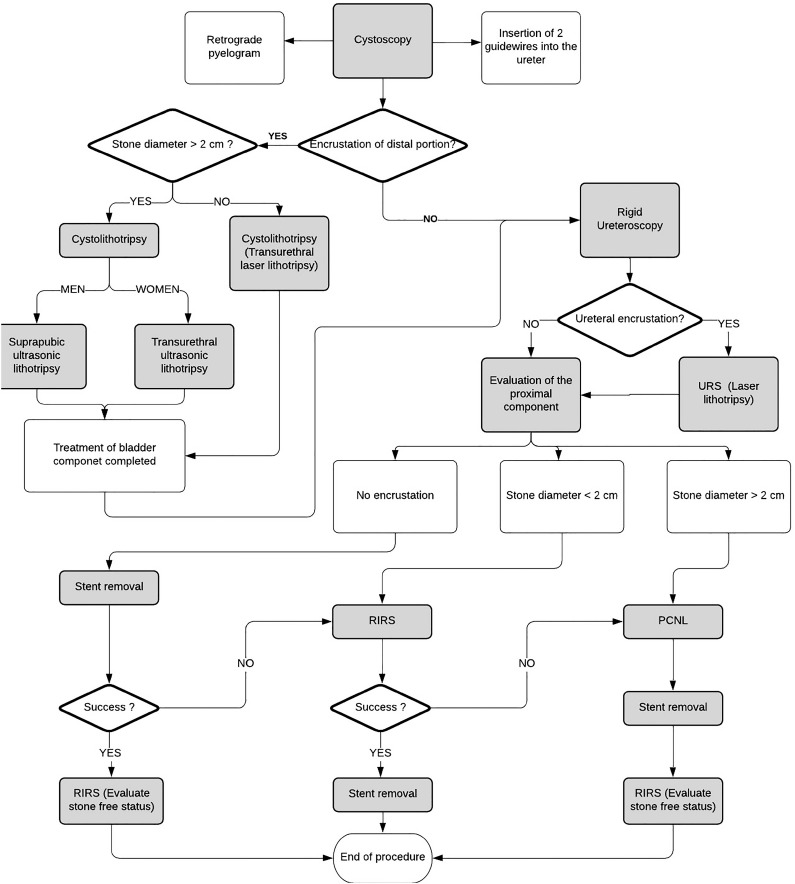
Surgical technique: Flow chart diagram.

Our approach was as follows (Figure-2): Starting with cystoscopy, contrast was retrogradely injected into the upper urinary tract using a 6Fr single lumen ureteral catheter. Two 0.038” hydrophilic guidewires were radioscopically placed in the ureter. We then addressed the stone encrustation of the vesical portion of the stent by performing cystolithotripsy (CLT) using either laser (Holmium YAG laser) or ultrasonic energy (CyberWand Dual Ultrasonic Lithotriptor®, Gyrus, Olympus, Japan). If the distal stone maximum diameter was less than 2cm, transurethral laser lithotripsy was performed. Alternatively, if the distal stone maximum diameter was more than 2cm, transurethral lithotripsy, using a nephroscope and an ultrasonic probe, was performed in women; while in men, a laparoscopic 10mm trocar was placed percutaneously 3cm above the pubic symphysis. The trocar was placed with a full bladder and a nephroscope was used through it to avoid risks of urethral injury and stenosis.

After treating the bladder component, a 6.5Fr rigid ureteroscope (Karl Storz®, Germany) was used to inspect the ureter in all cases. Ureteral stones were treated with a 200-µm laser fiber (Holmium YAG laser), with fragmentation settings such as 0.8-1.2J (pulse energy) × 4-10Hz (frequency) in a single pulse width (350µs). Forceps, graspers, and nitinol baskets were used for extraction of stone fragments and catheter. If, after this, the stent was successfully retrieved, a flexible 7.5Fr permanent ureteroscope was used to look for residual fragments in the kidney. If the stent was stuck, RIRS was performed preferably through a 11/13Fr, 35cm access sheath. When there were any failures to advance the access sheath parallel to the encrusted catheter, procedure was performed without it.

When the stone burden inside the kidney was greater than 2cm, percutaneous access was indicated. This was achieved by a urologist under biplanar fluoroscopic and ultrasound guidance. Usually, the subcostal percutaneous access, respecting the posterior axillary line, was single. The pelvicalyceal system was usually entered at the lower posterior calyx, and middle and upper calyx punctures were used when stones were present within these calyces. After the introduction of a 0.038” hydrophilic guidewire through an 18-gauge needle into the collecting system, tract dilation was performed using fascial dilators (double shot technique: numbers 10, 20, and 30Fr, sequentially), and a 30Fr Amplatz® sheath was placed. Nephroscopy was performed with a 26Fr rigid nephroscope (Karl Storz®, Germany), and stone fragmentation and suction were performed with an ultrasonic lithotripter.

An intraoperative stone-free status was verified with fluoroscopy, flexible nephroscopy and flexible ureteroscopy. At the end of the procedure, a 16Fr nephrostomy tube was placed in cases of bleeding, residual stones, solitary kidney, suspected pelvic injury, or multiple tracts. A 6Fr ureteral catheter was routinely left in place; in cases of a ureteropelvic junction with significant edema, extensive pelvic injury, or ureteral manipulation, a 4.8Fr x 26cm ureteral stent with external string was used instead. Ropivacaine 1% 20mL was injected into the tract for pain control, and an 18Fr Foley catheter was placed on the bladder (14).

### 

#### Endpoints

Surgical management (including both stent retrieval and urinary lithiasis treatment), number of interventions to achieve stone-free status, surgical time, hospital stay, postoperative complications assessed using the Clavien-Dindo classification (15), and residual stones were compared between groups according to the FECaL classification. The primary endpoint was the one-step removal of calcified stents, while the secondary endpoint was to compare groups in terms of postoperative outcomes. Success was defined as stone-free patients, meaning no fragments in the CT of the first postoperative day.

### Statistical Analysis

ANOVA was used to compare numerical variables between groups, and the Mann-Whitney, or Chi-square tests were used to compare categorical variables between groups. SPSS software version 19.0 for Windows (SPSS Inc.) was used for statistical analysis, and a p-value <0.05 was considered statistically significant.

## RESULTS

We enrolled 50 patients, all of whom had a functioning kidney after evaluation with contrast CT and scintigraphy. The mean follow-up was 2.9±1.4 years (1.7-4.6 years). Patients were divided into groups according to the FECal classification, and were comparable in terms of age (45.7±13.9 years), sex, laterality, BMI (26.3±4.9kg/m2), comorbidities, ASA, reason for stent passage, and indwelling time (Table-1). All stents were successfully removed in a single session using a combined endourological approach. The mean surgical time, stone-free rate, and complication rate were 115.5±53.5 minutes, 66%, and 22%, respectively (Table-2).

**Table 1:** Demographic data of patients presenting encrusted ureteral stents according to the FECal Classification.

		Grade I	Grade II	Grade III	Grade IV	Grade V	Total	p - value
Number of patients		11	8	10	11	10	50	
Age (Years, mean ± SD)		43.7 ± 12.5	47.7 ± 12.7	42.8 ± 9.9	47.1 ± 18.5	47.7 ± 15.8	45.7 ± 13.9	0.71
Gender (M:F)		27.3%: 72.7%	25%: 75%	40%: 60%	45.5%: 54.5%	50%: 50%	36%: 64%	
**Side**								
	Left	45.5%	25%	40%	54.5%	60%	46%	
	Right	54.5%	75%	60%	45.5%	40%	54%	
BMI (Kg/m^2^, mean ± SD)		26.9 ± 4.5	28.8 ± 6.1	26.0 ± 3.9	24.1 ± 3.8	26.2 ± 6.53	26.3 ± 4.9	0.89
**ASA classification**								
	I	54.5%	37.5%	60%	54.5%	20%	20%	0.35
	II	27.3%	62.5%	40%	18.2%	60%	60%	
	III	9.1%	--	--	27.3%	20%	20%	
	IV	9.1%	--	--	--	--	--	
**Reason for Stent Placement**							–	
	Urinary sepsis	4 (36.4%)	4 (50%)	4 (40%)	2 (18.2%)	3 (30%)	17 (34%)	0.47
	Acute kidney injury	2 (18.1%)	3 (37.5%)	1 (10%)	3 (27.3%)	2 (20%)	11 (22%)	
	Pain and Hydronephrosis	2 (18.1%)	0	4 (40%)	3 (27.3%)	4 (40%)	13 (26%)	
	Definitive treatment non-available	0	1 (12.5%)	0	1 (9.1%)	1 (10%)	3 (6%)	
	Other	3 (27.3%)	0	1 (10%)	2 (18.2%)	0	6 (12%)	
Indwelling Stent Time (Months, mean ± SD)		7.3 ± 3.0	26.1 ± 10.0	13.8 ±10.7	30.1 ± 20.1	32.8 ± 23.2	21.7 ± 13.4	0.11

**Table 2:** Patients operative and peri-operative data.

		Grade I	Grade II	Grade III	Grade IV	Grade V	Total	P - value
Stone burden (cm^3^, mean ± SD)		288.4 ± 252.4	489.0 ± 385.7	1056.0 ± 842.4	8044.1 ± 4779.2	10699.3 ± 9497.8	4262.4 ± 3236.7	0.027
PCNL		0	62.5%	70%	81.9%	100%	64%	0.004
ULT		100%	100%	40%	54.5%	80%	60%	< 0.001
CLT		54.5%	0	0	100%	100%	62%	0.281
Surgical Time (Min, mean)		65 ± 32	100 ± 25	110 ± 64	138 ± 74	164 ± 67	115.5 ± 53.5	0.006
Number of Procedures to Stone Free Status		1.36 ± 0.9	2.25 ± 1.5	2.7 ± 2.0	1.28 ± 1.2	2.25 ± 1.5	1.92 ± 1.4	0.687
Hospital Stay (Days, mean ± SD)		4.4 ± 2.6	2.60 ± 1.8	2.9 ± 1.8	5.7 ± 2.4	7.0 ± 2.1	4.2 ± 2.5	0.141
Blood transfusion		0	0	0	9.1%	0	2%	
**Clavien classification**								
	Without Complication	81.8%	62.5%	80%	72.7%	90%	78%	0.541
	I	9.1%	37.5%	20%	9.1%	0	14%	
	II	0	0	0	0	0	0	
	III a	9.1%	0	0	0	0	2%	
	III b	0	0	0	9.1%	10%	2%	
	IV a	0	0	0	9.1%	0	2%	
	IV b	0	0	0	0	0	2%	
**Success (Stone-free)**		90.9%	87.5%	60%	54.5%	50%	66%	0.081
**Stone Analysis**								
	Calcium Oxalate	81.8%	100%	50%	45.5%	60%	66%	
	Struvite	0	0	50%	54.5%	20%	26%	
	Calcium phosphate	9.1%	0	0	0	0	2%	
	Mixed	9.1%	0	0	0	20%	6%	

Stone burden was higher for grade IV and V (8.4±4.8 and 10.6±9.5cm3) in comparison to all other grades (p=0.027). PCNL was the most common intervention for grades IV and V (81.8% and 100%, respectively), was not performed in grade I, and regularly performed in grade II (62.5%), as these encrustations were usually minor and not located in the kidney (p=0.004). URS was commonly used for ureteral stent encrustation, especially in groups with lower stone burden (grades I and II). Furthermore, the operation time was higher in groups III to V (110±64 and 164±67 min) (p=0.006), as PCNL was commonly performed in these cases (Table-2 and Table-3). RIRS was performed in all cases (Figure-2), preferably after stent removal to check stone free status. In 4 cases (8%) access sheath was used parallel to the stent.

**Table 3:** Procedures according to FECal Classification.

	Grade I	Grade II	Grade III	Grade IV	Grade V
**URS + RIRS**	5 (45.5%)	3 (37.5%)	3 (30%)	0	0
**PCNL + RIRS**	0	0	6 (60%)	0	0
**PCNL + URS + RISRS**	0	5 (62.5%)	1 (10%)	0	0
**CLT + URS + RIRS**	6 (54.5%)	0	0	2 (18.1%)	0
**CLT + PCNL + RIRS**	0	0	0	5 (45.5%)	2 (20%)
**CLT + URS + PCNL + RIRS**	0	0	0	4 (36.4%)	8 (80%)

Complications occurred in 22% of patients. Most (14%) were minor complications (fever, pain, emesis), 1 case required radiological intervention (urinoma), and 3 other cases required critical care for sepsis; the organisms in question were enterobacteriaceae such as Klebsiella pneumoniae and Escherichia coli. Reason for missed stents in all cases was limitations on the public health system.

The number of procedures, hospital stay, blood transfusion, complications, and stone analysis were similar between groups. The stone-free status was not significantly different between groups but was lower in grades III to V compared to grades I and II (Table-2). Residual fragments treatment decisions were made individually according to stone size, location, and composition.

## DISCUSSION

Stent encrustation is one of the most serious complications of polyurethane double-J stents (2). Multimodal endourology forms the cornerstone of therapy for heavily encrusted retained stents (3-5). Some investigators have reported high success rates in managing calcified stents using endourologic techniques in a single anesthetic setting (9, 16-18); however, it is common to need multiple sessions to successfully render the patient stent and stone-free, depending on which modalities are used (4, 7). In this series, all stents removed were made of polyurethane.

Imaging plays a pivotal role in evaluating the patient and determining appropriate surgical management of the encrusted and retained stent. Furthermore, quantifying the stone burden associated with encrustation has prognostic significance, and contrast CT has been shown to help evaluate renal function (7). A poorly functioning kidney with significant stone burden may be better suited for nephrectomy rather than multiple procedures to eliminate all stones (2).

If no encrustation is visible on plain radiography, removal of the stent in a retrograde fashion may be attempted. Ideally, fluoroscopy should be available to determine if there is uncoiling of the proximal curl during removal, because this may be a site of resistance. If there is any resistance during the at tempt at cystoscopic removal, one should stop immediately because the risk of stent fracture or ureteral injury cannot be ignored. In all cases, ureteroscopy was performed after the placement of two guidewires, leaving one as a safety guidewire.

Calcifications along the ureteral component of the stent can be managed with retrograde ureteroscopy and laser lithotripsy. In our experience, the ureter always accommodated the ureteroscope after placing two guidewires. The encrustations usually affect the stent circumferentially, but in our cases the stent was not attached to the ureter. However, in a few cases, a prominent inflammatory reaction to the stent or to the initial stone causing symptoms that motivated the stent passage was observed. In such cases, surgeons should be extra-careful because pushing or pulling maneuvers can easily perforate or even avulse the ureter. Only small fragments are obtained by laser breakage of encrustations in a circumferential fashion, and the risks of ureteral injury are diminished.

For proximal ureteral stent encrustations in men especially and renal stent encrustations of less than 2cm, flexible ureterolithotripsy with a laser is indicated. For larger stent encrustations (more than 2cm), percutaneous lithotripsy is the preferred primary approach. In the case of simultaneous large proximal and distal encrustations, the main advantage of PCNL in the Galdakao-Valdivia supine position is to address both encrusted ends (proximal or distal) (8). ESWL can be an option in cases of proximal encrustation and lower stone burden, but as monotherapy may not be appropriate or recommended (18). In the current scenario the miniaturized PCNL is also an option, but the lower success rates on higher stone burden and the risk of elevated intra renal pressure lead us to believe that standard PCNL can have a safer profile in this situation (19).

Our approach was effective and suitable for patients. Furthermore, it offers the advantages of reduced patient handling, the requirement of a drape only once, the ability to perform simultaneous PCNL and ureteroscopic procedures, better control of the airway during procedures, and the ability of a surgeon to perform PCNL while sitting. To the best of our knowledge, this represents the largest experience by a single surgeon. Bostanci et al. (16) reported treatment of 19 patients using a single multimodal approach and a low rate of complications, but did not describe their results. Ulker et al. (17) treated 17 patients with a 58.9% stone-free rate in a single procedure, however, they were evaluated with KUB. Given that treatment is generally difficult, with potential hazardous complications (18, 20, 21), we believe that these cases should be referred to high volume endourological centers with experienced staff.

Compared to other grading systems for encrusted stents, the FECal classification system developed by Acosta-Miranda et al. incorporate both stone location and size (3). It is simple to utilize but, limited in that it was developed with a small sample size of nine patients. The KUB grading system (22) differentiates between stone burden involving the proximal versus the distal coil of the stent, which has important implications for surgical complexity, but has a complex and longer application.

With regards to the limitations of our study, it was not randomized, and did not include a control group. The lack of a significant difference in the complications and success may be due to the low statistical power (e.g., small sample size) rather than the absence of a difference. However, the study strengths may compensate for these limitations. Our study included a large number of patients compared to other series. The demographic data were similar in both groups; all patients had pre- and post-operative CT scans, and a complete prospective database was utilized in order to reduce the chances of bias on the similarity of the outcomes regarding positioning.

In the current study, we provided suggestion for the standardization of an efficient treatment of encrusted ureteral stents in a single-session procedure, that can be validated by a multicenter trial. Prevention is defined as the best treatment for encrusted stents. Measures such as control of stent period, encourage referral to high volume centers, development of new stents and new technologies should be applied (23, 24). However, if this dramatic situation occurs, the FECal classification is a good tool to predict outcomes of the cases, and the proposed approach seems to be a rational option for treatment.

## CONCLUSIONS

The endoscopic combined approach with the patient in the Galdakao-modified Valdivia supine position, is a safe and feasible technique that allows removal of retained and encrusted stents in a single procedure. The FECal classification of the encrusted stones seems to be useful for surgical planning.

## ABBREVIATIONS

FECal classification: forgotten encrusted calcified classification
BMI: body mass index
ASA: American Society of Anesthesiologists Physical Status Classification System
PCNL: percutaneous nephrolithotomy
ECIRS: endoscopic combined intra-renal surgery
CT: computed tomography
URS: ureterolithotripsy
RIRS: retrograde intra-renal surgery
CLT: cystolithotripsy
ESWL: Extracorporeal shock wave lithotripsy
KUB: kidney-urinary-bladder graph
